# Model-based Respondent-driven sampling analysis for HIV prevalence in brazilian MSM

**DOI:** 10.1038/s41598-020-59567-2

**Published:** 2020-02-14

**Authors:** Olivier Robineau, Marcelo F. C. Gomes, Carl Kendall, Ligia Kerr, André Périssé, Pierre-Yves Boëlle

**Affiliations:** 1 INSERM, Sorbonne Université, Institut Pierre Louis d’Épidémiologie et de Santé Publique, F75012 Paris, France; 2 Service Universitaire des Maladies Infectieuses et du Voyageur, Tourcoing, France; 3 Fundação Oswaldo Cruz (Fiocruz), Programa de Computação Cientifica, Rio de Janeiro, Brazil; 4 Department of Global Community Health and Behavioral Sciences, School of Public Health and Tropical Medicine, Tulane University, New Orleans, Louisiana, USA; 5 Department of Community Health, School of Medicine, Federal University of Ceará, Fortaleza, Brazil; 6 Fundação Oswaldo Cruz (Fiocruz), Escola Nacional de Saúde Pública Sergio Arouca (ENSP), Departamento de Ciências Biológicas, Rio de Janeiro, RJ Brazil; 7 Sorbonne Université, INSERM, Institut Pierre Louis d’Epidémiologie et de Santé Publique, AP-HP, Hôpital Saint-Antoine, Santé publique, F75012 Paris, France

**Keywords:** HIV infections, Epidemiology

## Abstract

Respondent Driven Sampling study (RDS) is a population sampling method developed to study hard-to-reach populations. A sample is obtained by chain-referral recruitment in a network of contacts within the population of interest. Such self-selected samples are not representative of the target population and require weighing observations to reduce estimation bias. Recently, the Network Model-Assisted (NMA) method was described to compute the required weights. The NMA method relies on modeling the underlying contact network in the population where the RDS was conducted, in agreement with directly observable characteristics of the sample such as the number of contacts, but also with more difficult-to-measure characteristics such as homophily or differential characteristics according to the response variable. Here we investigated the use of the NMA method to estimate HIV prevalence from RDS data when information on homophily is limited. We show that an iterative procedure based on the NMA approach allows unbiased estimations even in the case of strong population homophily and differential activity and limits bias in case of preferential recruitment. We applied the methods to determine HIV prevalence in men having sex with men in Brazilian cities and confirmed a high prevalence of HIV in these populations from 3.8% to 22.1%.

## Introduction

Respondent-driven sampling (RDS) is a method to sample hard-to-reach populations such as injecting drug users, men who have sex with men (MSM), and sex workers^1^. It uses chain-referral sampling, building on the underlying contact network for recruitment of participants. RDS starts by selecting *seed* individuals from the population of interest. They receive a fixed number of coupons to distribute to individuals in their contact network who meet certain eligibility criteria. In turn, individuals receiving a coupon recruit new participants among their contacts, leading to successive recruitment waves until the target number of individuals for the survey is reached^2^. A drawback of the method is that the final sample is not representative of the target population, introducing bias in naïve estimates of, say, prevalence.

Statistical procedures using generalized Horvitz-Thompson estimators can reduce biases^3,4^. In those, weights are computed according to referral patterns, estimated network size, number of ties between subgroups of interest^5^, differences in the number of partners declared by participants^6^ or homophily in chain referrals^7^. Weights can also be computed using bootstrap procedures^8^. Yet, numerous issues affect the reliability and validity of RDS estimates because several hypotheses are required^3,9–11^: (1) the population size need to be large compared to the RDS sample; (2) sampling must occur with replacement; (3) population homophily should be weak; (4) seeds should be selected at random.

Recently, the network model-assisted (NMA) method has been shown to increase the robustness in prevalence estimation from RDS data with respect to these assumptions^11^. In this approach, characteristics of the RDS data are used to simulate population networks that resemble the source population. The simulated networks conform to degree distributions and other individual characteristics of participants, but, more importantly, allow pair characteristics such as homophily between nodes with similar characteristics, to be taken into account. In simulation studies, the NMA approach fared better than other methods to estimate HIV prevalence in RDS data even in the case of population homophily according to HIV status, when seeds were selected according to HIV status; when the RDS sample size was large relative to the underlying population and the degree distribution changed according to the outcome of interest^11^. The approach however requires knowing the HIV serostatus of all partners of individuals in the RDS sample even those who were not included in the study. This is unlikely to be the case for most RDS studies as serostatus is often determined only in participants. Here, we develop the use of “known patterns” for applying NMA method in this situation^12^. An additional concern is that homophily may not be easily ascribed to the underlying population or to preferential recruitment in RDS data^13^. Assumptions of no preferential recruitment were made in the original method and it is unknown whether accounting for “homophily” of non-specified origin may improve prevalence estimates.

Here, we first show that an iterative approach for applying NMA method leads to significant performance improvement in the case of non-random seed selection in an underlying network with strong homophily and differential activity. We next examine the effect of preferential recruitment on prevalence estimates. We finally compare NMA methods to other methods on RDS data from MSM in Brazil to estimate HIV prevalence.

## Material and Methods

We first recall the principle of weighted estimates for estimating HIV prevalence with RDS data and describe an extension to Giles’ method for NMA prevalence estimation. Then, a simulation study is presented to investigate the performance of this estimator with three known sources of bias: seed selection, seed dependence, and population homophily. We next investigate the presence of preferential recruitment in the RDS. Finally, we apply the methods to data collected in an RDS study of MSM in Brazil.

### Current methods for estimating HIV prevalence in RDS data

An RDS sample for HIV prevalence yields a collection of random variables (y_i_) corresponding to the serological status of individuals, with y_i_ = 1 for HIV positive and 0 otherwise. Horvitz-Thompson estimates of the prevalence are computed as $\hat{\pi }=\frac{{\sum }_{i=1}^{n}{w}_{i}{y}_{i}}{{\sum }_{i=1}^{n}{w}_{i}}$ where weights w_i_ correspond to the inverse probability of sampling. Methods differ in the choice of w_i_: inverse degree for RDS-I^5^, with further modifications in RDS-II^6^ and the SS (successive sampling) estimator^8^. The tree bootstrap method allows for another way to compute weights^14^. These approaches are implemented in RDS Analyst, the RDS package for the R Software^15^, and the RDS treeboot package^14^.

In the NMA method, the computation of weights requires network simulation. This makes it possible and easy to include individual characteristics in modeling contacts, such as infectious status^11^, and to account for population homophily, i.e., the differential probability of making links with other individuals based on a specific characteristic. In this approach, population homophily, for example for HIV serostatus, can be defined as the ratio of the observed number of links between discordant HIV serostatus to the number of such links expected by chance^15^ where values smaller than 1 correspond to more links than expected between individuals with identical serostatus. This definition corresponds with the parameters used in exponential random graph models (ERGM).

In the original NMA method, it was assumed that the serostatus of all contacts, whether included or not in the RDS, had been reported by each participant. This allowed estimating homophily in the following algorithm for seroprevalence:

**Table:** 

Initialization: • compute degree distribution d°_Y_ in RDS data according to serostatus Y • compute initial weights w_i_ = w°_i_ according to an arbitrary RDS method (here SS method) • compute homophily h° from RDS data
Repeat: Step 1: Simulate M1 networks with degree distribution (d°i) and homophily h° Step 2: Obtain M2 RDS samples from each simulated network with seeds characteristics as in the original RDS data and with the same number of participants. Step 3: Compute weights distribution wi by degree and serostatus y based on the simulated RDS averaging over the M1 * M2 RDS samples
Until: • Weights convergence or 5 iterations
Output: • Compute seroprevalence $\hat{\pi }=\frac{{\sum }_{i=1}^{n}{w}_{i}{y}_{i}}{{\sum }_{i=1}^{n}{w}_{i}}$ using final weights

Confidence intervals were obtained by the parametric bootstrap using the weights in the last iteration of the procedure a network was simulated using the last weights, RDS was sampled from the network and prevalence estimated from this data^11^. We developed a method to accelerate the Network-Model Assisted method by using configuration network (see supplementary information).

### Working with limited information on homophily in the network-assisted method

However, in our experience and the data presented below, participants are unlikely to know or provide information on the serostatus of all of their contacts. Often, accurate information on HIV serostatus about sexual partners is only available in those who were recruited in the RDS study. We propose the “NMA-Iter” approach in which imputation of the missing information is first made using the fraction of HIV seropositive observed in direct partners and then updated is updated with other quantities at each iteration. In other words, we start assuming that if 1 out of 3 of the recruited contacts were HIV seropositive for a respondent, then on average 1/3 of all of his contacts were HIV positive, and update this information using the degree distribution computed at each iteration as described in the following algorithm:

**Table:** 

Method NMA-Iter Initialization: • Initialisation steps of NMA • For each individual i in the sample set Ii = ki * Pi (infected partners) and Ui=ki* (1-Pi) (uninfected partners) where Pi is the percentage of HIV seropositive recruited partners and ki reported the number of partners. • Compute Homophily: H = 2 * p_w_ * degree_whiv−_ * (1-p_w_)*degree_whiv+_/(degree_woverall_ * degree_heterophilic_) Where p_w_ is the overall prevalence of HIV infection (weighted mean of x_i_), and degree_overall,w_, degree_hiv-,w_, degree_hiv+,w_, and degree_heterophilic,w_ are, respectively, the mean degree in the overall population (weighted mean of k_i_), in those HIV− (weighted mean of k_i_ when y_i_ = 0), HIV+ (weighted mean of k_i_ when y_i_ = 1) and that of heterophilic links (weighted mean of y_i_ U_i_+ (1 − y_i_) I_i_). All these quantities are post-calibrated with current weights w_i_
Repeat: Step 1–3 of NMA method Step 4: • Compute Homophily as above with current weights. • Simulate a network Nw with degree distribution according to serostatus D(s) and Homophily H. • From Nw, compute Y(d,s) the distribution of the number of HIV+ partners in individuals with status s and degree d. • For each patient in the RDS dataset, sample I_i_ from Y(di, si), compute U_i_ = d_i_ − I_i_ Loop and stop as in NMA method

### Simulation study

#### Network and RDS simulation

To investigate the properties of the new proposed estimation procedure (NMA-Iter), we conducted simulation experiments, obtaining RDS data in a population where HIV prevalence was fixed at 20%. We varied the following parameters in the population:

- Population size (Nw). Populations were either 10000 or 1000, so that an RDS sample of size 400 corresponded to either a small (4%) or large (40%) portion of the whole population.
- Differential Activity. DA is defined as the ratio of the average number of partners in HIV seropositive participants and others. It allows simulating differential recruitment according to the characteristic of interest, here HIV serostatus. DA = 1 means no difference, while DA = 2 corresponds to twice as many partners for HIV positive participants.
- Homophily. We finally varied homophily according to the characteristic of interest. Recruitment could be irrespective of serostatus (Homophily = 1) or lead to highly clustered data with twice as many partners of the same status than expected (H = 2).

These characteristics defined six scenarios, for which 150 networks were simulated in each case. Network simulations were performed using the method described in the appendix and exponential random graph models (ERGM) in the R package statnet^16^.

RDS samples of size 400 were sampled in each simulated network, using three coupons per participant and an (independent) participation rate of 50%. We started recruitment with seven seeds chosen at random or with seven infected seeds selected, in both cases selected with probability proportional to the degree.

Results were compared graphically by boxplots of the bootstrap distributions. We also computed mean squared errors (MSE) to compare overall errors, including bias and variance of the estimators, for the NMA, NMA-Iter and SS methods.

#### Effect of preferential recruitment (PR)

To investigate the impact of PR in prevalence estimates we obtained RDS samples in the absence of preferential recruitment, then increasing the probability for a partner with the same serostatus to be recruited by 1.5 compared to a partner with discordant serostatus. These situations are described as “No PR” and “+50% PR”.

#### Comparison between methods

We first computed prevalence estimates in the simulated RDS data using the NMA method, assuming full information on homophily. We then computed prevalence estimates using NMA-Iter with limited information on homophily. We used boxplots to show the corresponding distributions.

We then compared estimates from NMA-Iter to estimates obtained using classical RDS analysis methods (RDS-I, RDS-II, SS) in several situations with varying (1) serostatus of seeds (random or infected), (2) RDS sample size relative to the underlying network: 40% (size 1000) or 4% (size 10000) and (3) magnitude of homophily and DA (1 or 2 for both).

#### Investigating seed selection

Convergence plots were used to visualize the effect of initial waves on the stability of the final estimates^3^. In this plot, the prevalence estimates are plotted as a function of the accruing number of waves of recruitment. It shows both the effect of initial seed selection and that of sample size.

As it is also customary to drop initial waves in prevalence computations to reduce dependence on the *seeds*, we also show “drop-first convergence plots” where prevalences are computed using all waves but the first ones (i.e., dropping the first, then the first and second, and so on).

We visually inspected convergence plot, and drop-first convergence plots for the SS and NMA-Iter for RDS sampled in networks with no homophily and low DA and in networks with high homophily and high DA. These plots are shown starting at wave 3 and stopping at the last wave of recruitment which might vary from one simulation to another.

### Application to HIV prevalence in Brazil

Several RDS studies have been carried out in Brazil in populations with high HIV prevalence^17–23^. Here, we re-analyzed the nationwide survey conducted in 2009 to estimate HIV prevalence in MSM^23^. The survey was conducted in ten Brazilian cities, as described in Table 1.

**Table 1:** characteristics of the RDS in 10 cities of Brazil.

City	Sample size	Number of waves	Number of recruited by waves	Number of seeds	Number of infected seeds	Homophily estimate using NMA-Iter
Manaus	848	20	42	10	0	0.55
Recife	351	12	29	10	3	1.01
Salvador	383	20	19	18	2	1.34
Brasilia	344	17	20	10	0	0.46
Campo Grande	351	17	21	7	1	1.71
Belo Horizonte	274	15	18	21	5	0.50
Rio de Janeiro	357	12	30	13	2	1.52
Santos	304	16	19	12	2	1.08
Curitiba	337	13	26	32	2	0.83
Itajai	310	13	24	15	5	0.93

We applied seven methods to estimate HIV prevalence in each city. For the NMA method, we used a population size of 10000 MSM in each city (a sensitivity analysis showed that estimates did not change significantly above 5000 individuals). We considered that two methods disagreed for a given city when at least one-point estimate was not within the confidence intervals of the other for that same city.

In order to investigate the differences among estimates, we looked at seeds characteristics, population size and examined the convergence plots^3^ and drop-first convergence plots.

### Ethics

Data from The Brazilian RDS survey was approved by the Brazil National Ethical Research Committee (Comissão Nacional de Ética em Pesquisa, CONEP # 14494). Informed consent was obtained for all participants who signed consent forms for participation to the survey and later use of results^23^. All methods were carried out in accordance with relevant guidelines and regulations^1^. No additional approval was necessary for the current research work.

## Results

### Simulations

#### Performance of the NMA modified method

**Figure 1:**
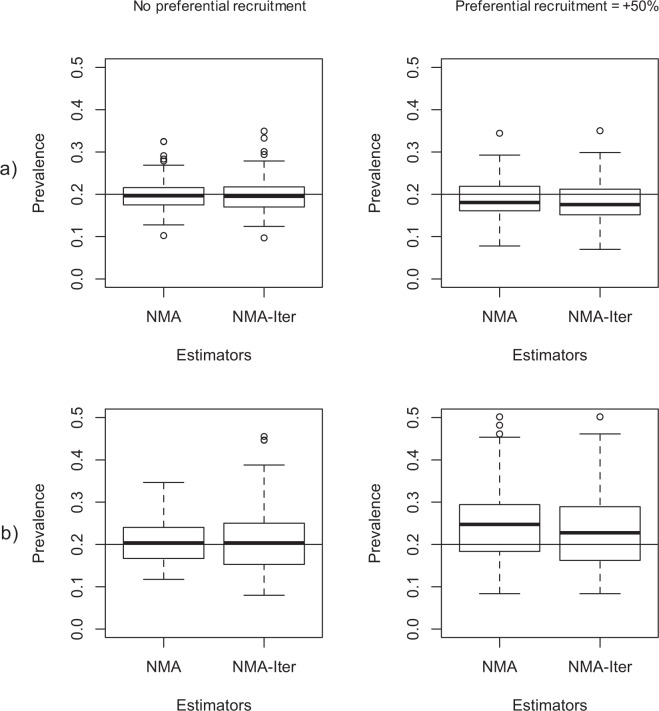
Prevalence estimates with network model assisted method and effect of Preferential recruitment. NMA is the original method requiring full information on serostatus of contacts, NMA-Iter use only serostatus of reported contacts. Preferential recruitment were fixed at no preferential recruitment and +50% of preferential recruitment (left to right). Simulation were performed in networks of size 10000 and: (**a**) population homophily at 1 and DA at 1; (**b**) population homophily at 2 and DA at 2.

The NMA-Iter method allowed estimating prevalence even when serostatus information was limited to RDS participants, with estimates close to the target value (20%; Fig. 1). The results very similar to the original NMA method. The variability of the estimates from the NMA-Iter method did not increase compared to the original NMA, with similar MSE in both cases (1.51 × 10^−3^ and 1.59 × 10^−3^ for no homophily nor DA; 2.51 × 10^−3^ and 2.55 × 10^−3^ for strong homophily and DA). Additional preferential recruitment led to an increase in bias for both the NMA and the NMA-Iter methods, even though the true prevalence value remained in the confidence intervals for the simulated RDS sample sizes (Fig. 1).

The estimated homophily was close to the nominal in the case of no homophily in the simulated population (H = 1) and no differential activity and no preferential recruitment (estimated value 0.96 [0.87–1.05]). Bias occurred with deviations from the random situation, with downward bias for increasing differential activity (estimated homophily 0.90 [0.81–1.02] for DA = 2 and no PR), and upward bias with preferential recruitment and no DA (1.09 [1.01–1.18] for PR + 50%). With population homophily (H = 2) in the simulated population, this was generally underestimated in the NMA method (estimated homophily = 1.45 [1.29–1.73]), and the estimate increased in case of additional preferential recruitment (1.50 [1.16–2.00] for + 50% PA).

**Figure 2:**
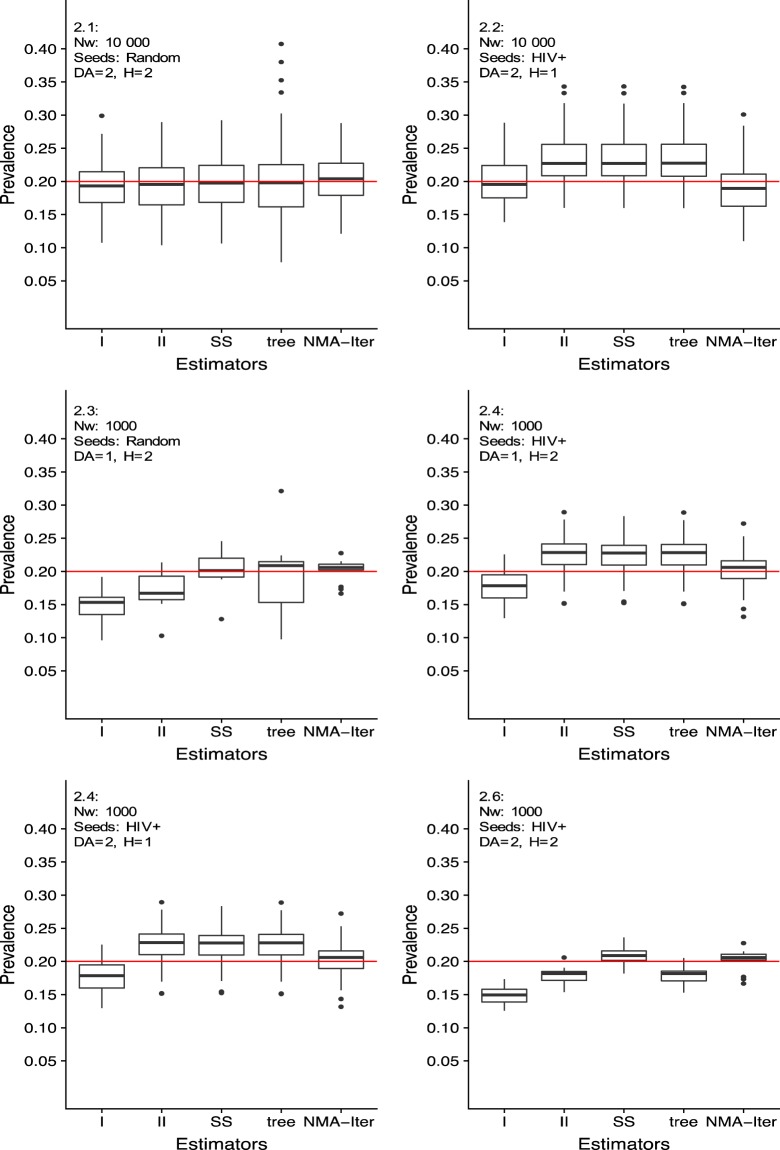
Prevalence estimates using different network patterns in networks of different size. Nw: network size, DA: degree activity, H: Homophily. DA can be at 1 or 2 and H can be at 1 or 2, Nw can be at 10000 or 1000. RDS are of size 400. Preferential recruitment is at 1.

The seroprevalence estimates in the NMA-Iter is shown in Fig. 2 along with other methods. When the population size was large relative to the RDS and seeds were selected at random, all methods yielded similar results even with high homophily and DA. (Fig. 2.1). The bias introduced by selecting only HIV + seeds was the smallest for the RDS-I and NMA-Iter methods (Fig. 2.2). When populations were small (1000 individuals, RDS including 40% of population size), and when seeds were chosen at random, the NMA-Iter method, SS method and tree bootstrap method performed the best under conditions of high homophily and high differential activity (Fig. 2.3). The NMA-Iter remained unbiased when all seed were HIV + in networks with high homophily or DA (Fig. 2.4 and 2.5). Finally, the NMA-Iter method provided the best results for small populations, high homophily and DA, and use of all HIV + seeds (Fig. 2.6).

**Figure 3:**
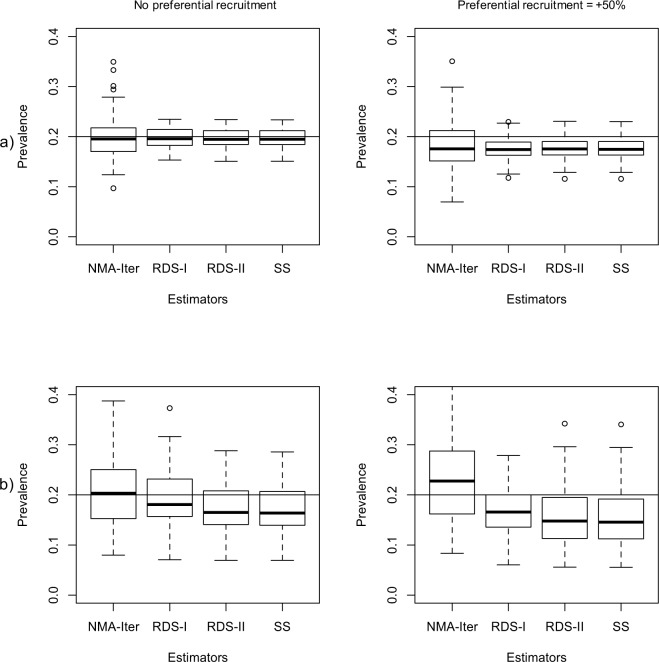
Comparison of different methods to estimates prevalence in RDS with different preferential recruitment. Preferential recruitment were fixed at “No PR” and “+50% PR” (left to right). Simulation were performed in networks of size 10000 and: (**a**) homophily at 1 and DA at 1; (**b**) homophily at 2 and DA at 2.

The presence of preferential recruitment had an impact on seroprevalence estimates for all methods tested (NMA-Iter, RDS-I, RDS-II, SS) (Fig. 3). The bias in the NMA-Iter method remained smaller than in other methods but there was a corresponding increase in variance of the estimates. In the most extreme situation (H = 2, DA = 2, PR = 1.5), the MSE was 12.3 × 10^−3^ for NMA-Iter and 4.8 × 10^−3^ for the SS method.

#### Comparison of SS and NMA-Iter in convergence and drop-first convergence plot

**Figure 4:**
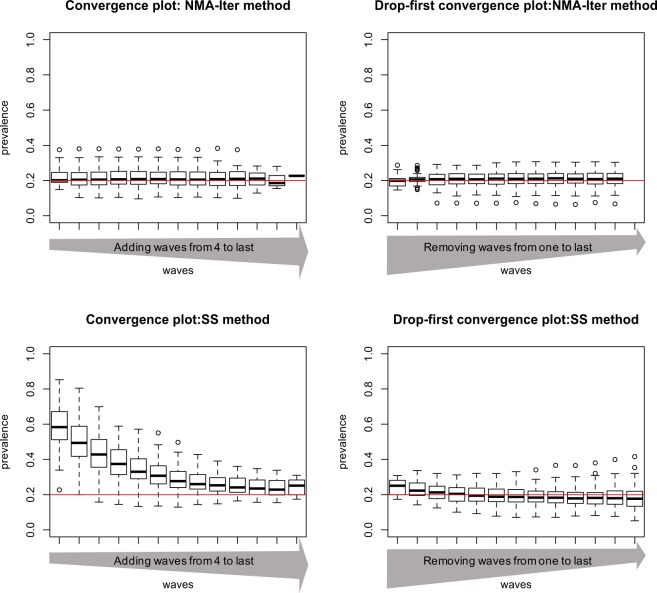
Convergence plot and drop-first convergence plot using SS method (upper part) and NMA-Iter (lower part) in network with high homophily and high degree activity (H = 2 and DA = 2).

In networks combining differential activity and homophily, the convergence plots showed that the prevalence computed by the SS method was more sensitive to the choice of initial seeds than the NMA-Iter method, with estimates reaching a plateau only after the 10^th^ wave for the SS method and much quicker with the NMA-Iter method (Fig. 4). The drop-first convergence plots even showed that dropping early waves may introduce bias when using the SS method, while the NMA-Iter method remained satisfactory (Fig. 4).

### HIV prevalence estimation for Brazilian MSM

Table 2 shows the results for the ten cities using six methods. For most cities, point estimates were between 2 and 10% regardless of the method for estimation, with the exception of Rio de Janeiro and Brasilia where prevalence is around 20%. The results from the different methods did not agree in Recife, Campo Grande, Rio de Janeiro, Santos, Curitiba, and Itajaí (i.e., at least one pair of methods with one-point estimate falling outside the confidence interval of the other method). In Brasilia, variations were substantial for the different methods with correspondingly large CIs. In this city, the point estimate was on the order of 10% for RDS-I, RDS-II, and SS methods, while the Tree bootstrap and NMA-Iter returned a point estimate of around 20%.

**Table 2:** Prevalence of HIV in MSM in 10 Brazilian cities based on different RDS analysis methods and from from Kerr and al. previous work.

City	Previous HIV estimates (logistic regression)	RDS-I	RDS-II	SS	Tree	NMA-Iter
Manaus	8.3 [6–10.9]	8.0 [4.8–11.1]	8.0 [4.9–11.2]	8.0 [5.1–11.0]	6.9 [2.5–12.7]	7.4 [6.5–8.4]
Recife	5.2 [2.7–8.2]	4.5 [1.2–7.8]	5.0 [1.7–8.3]	5.0 [1.6–8.5]	4.5 [1.8–11.4]	5.7 [4.8–8.6]
Salvador	8.9 [5.5–12.7]	6.3 [3.8–8.8]	6.2 [1.8–10.7]	6.3 [1.9–10.6]	5.9 [2.2–13.9]	6.2 [5.5–9.4]
Brasilia	23.7 [16.6–31.5]	13.0 [3.1–22.8]	11.8 [1.8–21.7]	11.8 [4.9–18.7]	19.6 [8.3–34.3]	19.6 [8.0–25.0]
Campo Grande	6.7 [3.5–10.7]	3.3 [1.2–5.5]	3.3 [1.1–5.5]	3.3 [1.2–5.4]	5.0 [1.4–9.4]	4.8 [4.6–5.2]
Belo Horizonte	10.6 [4.2–16.1]	7.4 [0.8–14.0]	7.1 [0.6–13.6]	7.0 [0.8–13.3]	8.9 [3.0–28.0]	10.8 [7.1–12.1]
Rio de Janeiro	18.3 [10.9–24.9]	18.1 [9.6–26.5]	22.2 [13.9–30.4]	22.2 [13.2–31.1]	20.8 [5.0–40.2]	22.1 [17.9–25.5]
Santos	9.0 [5.2–13.5]	2.4 [−0.3–5.0]	3.3 [0.7–5.9]	3.3 [0.3–6.3]	4.0 [1.1–8.1]	3.6 [1.5–5.0]
Curitiba	18.9 [12.6–25.9]	6.3 [2.2–10.5]	7.6 [3.4–11.8]	7.6 [3.3–12.0]	12.8 [7.0–23.2]	9.8 [7.5–15.5]
Itajai	—	11.4 [2.2–20.5]	16.2 [7.0–25.4]	16.2 [6.6–25.8]	15.1 [8.0–25.4]	14.0 [13.4–33.5]

#### Seed selection

The proportion of HIV + seeds was high in comparison to the estimated prevalence in Recife (3/10) and Itajaí (5/15) (Table 1). Interestingly, the pattern of results in Itajai was similar to the situation in Fig. 2.4–2.5, with RDS-I yielding smaller prevalence than NMA-Iter and the 3 other methods yielding higher value. For Recife, the results were similar to case 2.6, with NMA-Iter yielding higher estimates than the others.

#### Population size

In Itajai, it seems that a large part of the MSM population was included in the RDS. Indeed, the RDS included 310 individuals, whereas the MSM population was estimated to range between 700 and 2000 (1 to 3% MSM in adults in a city with 7000 adults)^8^. Once again, the pattern of estimates looked like in Fig. 2 where the RDS fraction was large in the overall population.

#### Network structure

Homophily calculated using NA-Iter varied from 0.46 for Brasilia to 1.72 in campo grande (Table 1). As seen in the simulation part, non-random homophily might impact results from RDS I and RDS, even if seeds are selected at random. SS and treeboot are biased in the context of seed selection. This is in accordance with our results in Rio de Janeiro, Campo Grande, and Brasilia where homophily values were far from 1.

#### Convergence of estimates

**Figure 5:**
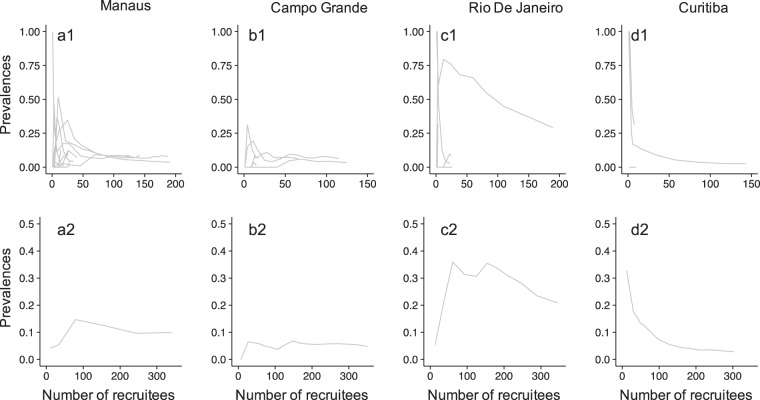
Graphical analysis of convergence. Top row: bottleneck plots showing for each city prevalence estimates evolution per seed during recruitment. Bottom row: convergence plots showing overall prevalence estimate during the recruitment process for each city. All estimates are based on the SS method.

**Figure 6:**
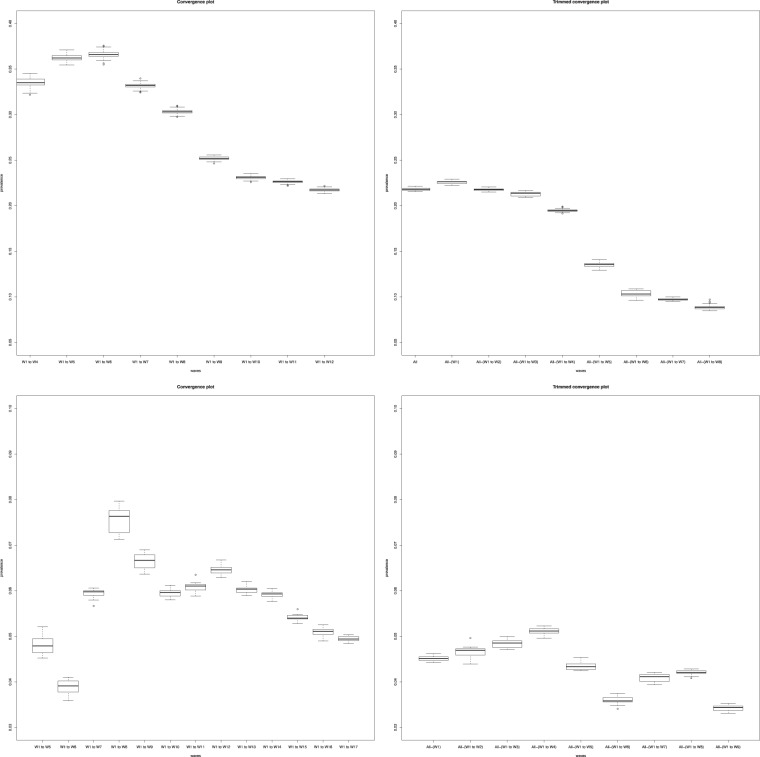
Convergence plot and drop-first convergence plot using NMA-Iter method for the city of Rio de Janeiro (upper part) and Campo Grande (lower part). W: Wave. Results from Rio De Janeiro confirmed the absence of convergence of the estimates. Results from Campo Grande show a convergence around 4% of prevalence. Impact of seed selection is visible up to wave 9.

Figure 5 shows convergence plots for four cities using the SS method. In Manaus, the graphical analysis demonstrated that the sample appeared to achieve convergence (Fig. 5a1,a2). In Campo Grande (Fig. 5b1,b2), convergence plots started with each seed indicated the possibility of distinctive sub-networks being selected, with little indication of convergence. However, in this city, the plots using the NMA-Iter methods suggested otherwise, with little change in the last waves (see Fig. 6).

Lack of convergence of estimates was demonstrated in Brasilia, Santos (not shown) and Rio de Janeiro (Fig. 5c1,c2). In Rio de Janeiro, even at the end of the sample, the slope of the convergence curve appears not to be leveling. It does create doubt that the estimated value is not the final value (Fig. 5c1,c2). This was confirmed for this city by using convergence plot and drop-first convergence plot using NMA-Iter method. Convergence plots showed substantial variations of the estimates before reaching waves 9. Drop-first convergence plot added value by showing that the last waves had still a strong impact on estimates, highlighting that equilibrium was not reached at the end of the RDS process (Fig. 6).

## Discussion

In this work, we investigated the use of the Network-Model Assisted method for prevalence estimation using RDS data with limited serostatus information. We found that an iterative method (NMA-Iter) had good properties when information on homophily was limited, even in the case of high differential activity, population homophily and non-random seed selection. An application of these methods to a Brazilian dataset allowed a critical assessment of the reported HIV prevalence among MSM.

### NMA-Iter method with respect to other methods

Network modelling is a promising method for estimating prevalence in RDS data as it allows using the characteristics of contact networks, however it requires information on the serostatus of all partners^11,15^. Here, we showed that prevalence estimates obtained when only the subset of individuals included in the RDS sample remained unbiased and did not increase the variability of estimates. This is of importance in practice, as only the serostatus of participants will be available in most RDS samples. An iterative update of all quantities provided better results than a single-step approach (results not shown). The NMA-Iter method kept the advantages already described for NMA compared to other methods in case of seed selection, small network size, population homophily and differential activity. It also compared favorably with the tree bootstrap methods in simulations.

Several assumptions are required to use RDS data for seroprevalence estimation. First, seeds should be selected at random in the population. In practice, one may end up including preferentially, for example, HIV infected individuals as seeds if they can be reached more easily when they seek care or to include a minimum number of cases when seroprevalence is low. The effect of initial seeds should vanish over waves of recruitment in RDS samples^8,10^, and this led to recommending dropping early waves before computing estimates. The “drop-first” plot showed that seed selection could affect some methods more heavily than others, was present in the SS method even when the first waves were discarded from the analysis and was much reduced in the NMA-Iter analysis. Using only infected individuals as seeds even led to good performance with the NMA-Iter method irrespective of the characteristics of both the population and the RDS size. The “drop-first” plot is an addition to diagnostic convergence plots^3^ for visual inspection of the effect of seed selection.

A second potential limitation is the size of the source population relative to the RDS sample, as the replacement assumption is more likely to be violated when the RDS sample amounts to a large part of this population. In the data we considered, the city of Itajaí may illustrate this situation, as the MSM population size is expected to range between 700 and 2000, while the RDS sample included 310. Simulations for small populations have shown that most methods are biased in this situation, even though it remains a minor contributor to overall bias^4,24^. This was confirmed for the SS estimator and the NMA-Iter that yielded consistent estimates even with sampling fraction as high as 40% of the population.

Last, there are effects of the underlying network structure, for example homophily according to HIV status which has been described among MSM, which affect the RDS data^25,26^. Identifying prevalence estimation methods that can account for it is of importance. The NMA-Iter method, as it estimates homophily from the RDS data and takes it into account for estimation, could reduce bias even with strong homophily and differential activity. These results were however shown with random selection among partners in the RDS data, i.e. no preferential recruitment. The main effect of preferential recruitment according to HIV status should be to increase homophily in the collected data. As both sources of homophily can not be told apart in RDS data, one could assume that estimating a single “working” homophily could preserve the characteristics of the NMA method^13^. In other words, not identifying the cause of homophily may be alright as homophily is a nuisance parameter in the NMA method rather than the target of estimation. Indeed, our simulations showed that the estimated “homophily” parameter in the NMA method changed with both preferential recruitment and population homophily, as it increased with increasing preferential recruitment and population homophily. The simulations showed that the unbiasedness observed with population homophily alone was not preserved in the case of preferential recruitment. NMA-Iter remained less biased than the other methods, but it was also more variable. It would be of interest to examine whether the partial identification reported in Crawford could further improve the NMA results in this respect^13^.

#### HIV prevalence in MSM in Brazil

Stigma and discrimination against MSM remain high in Brazil, especially among the poor and little educated populations^20,27,28^. This makes it difficult to obtain reliable HIV prevalence estimates among MSM and indicates RDS as a choice method for assessing prevalence^17^. Our analysis however showed that estimates changed according to the method and justified looking for those which are the less affected by characteristics of the underlying population. In this respect, we investigated seed selection, population size, recruiting practices, and network structure to find that the NMA-Iter method performed better than other methods. This gives ground to the consideration of its results as more relevant.

Yet, even with the NMA-Iter method, convergence to a well-defined HIV prevalence estimate did not occur in some cities. Too small sample size could be the reason. However, the 3 cities where this was the most apparent (Brasilia, Santos, Rio de Janeiro) did not have particularly small RDS sample size compared with other cities and, furthermore, simulations did not evidence cases of lack of convergence with more than 300 participants. It is more likely that the structure of the population may be at stake. Indeed, recent work indicated that clustering in the population could make prevalence estimates challenging, even after reaching a reasonable sample size^29,30^. As network structure cannot be known *a priori*, it is also reasonable to recommend analyzing RDS data during collection, to detect, as early as possible issues with the recruitment process which could lead to study failure^31^.

In this paper we have pursued Gile’s injunction to analyze and diagnose sources of bias in RDS studies^3^. The understanding of variation between estimates with different methods is essential to allow for meaningful comparisons. Finding the least biased estimate remains a crucial concern not just for statisticians, but for health authorities to follow the evolution of prevalence over time and to evaluate intervention effectiveness. In Brazil, comparable, national level RDS survey has been conducted among MSM, and been compared to the study reported here^32,33^. Controversy about the high seroprevalences reported, especially among young MSM reiterate the need for improved estimator methods satisfying both methodological and practical concerns to separate methodological and programmatic. How much of the differences found are due to surveillance implementation challenges, estimator methods, changes to the populations and behaviors classified as MSM, or changes in the underlying epidemic? Here we contribute to this discussion, demonstrating that network-assisted model, despite being more time consuming, was the least biased for sources explored in this study.

#### Limitations

The issues outlined in this work are known shortcomings for the RDS methods^3,4^. Although we have shown that the NMA-Iter method was better than most other methods, we cannot exclude that other unmodelled phenomena contribute to errors in the seroprevalence estimates.

## Conclusion

RDS remains at the core of HIV surveillance methods throughout the world for difficult to reach populations. Measuring changes in HIV prevalence is however limited by concerns regarding the variability of findings generated through RDS. The network-assisted model is a powerful method to estimate prevalence using RDS data that may be less biased than other methods. Its use should be encouraged, even if it still requires the use of specific software. These changes are all the more necessary when the effect of treatment as prevention or pre-exposure prophylaxis will have to be quantified in stigmatized populations as MSM in Brazil.

## Supplementary information

appendix.

## Data Availability

Data from the RDS survey are fully available upon request to the author, and are already available online, associated to previous publications.
