# Supplementary material for: Model-based Respondent-driven sampling analysis for HIV prevalence in brazilian MSM
Source: Sci Rep. 2020 Feb 14;10:2646. doi: 10.1038/s41598-020-59567-2 (PMC7021777; doi:10.1038/s41598-020-59567-2)
Supplement: Supplementary file 1 — appendix. [file 41598_2020_59567_MOESM1_ESM.docx]

**Model-based Respondent-driven sampling analysis for HIV prevalence in brazilian MSM**

Olivier Robineau, Marcelo F.C. Gomes, Carl Kendall, Liga Kerr, André Périssé, Pierre-Yves Boëlle

**Appendix**

***Accelerating the Network-Model Assisted method***

Step 1 of the NMA method and step 4 of the NMA-Iter require simulating networks with given target characteristics, including degree distribution and homophily. This can, for example, be done with Monte-Carlo sampling as implemented in the R package *ergm*. In our experience, though, convergence can be difficult to obtain with real data when extreme degree values are present. An alternative approach, when the number of target characteristics for the network is limited, is to build a configuration network satisfying the required homophily characteristics. Here, we first simulated a population of size N by sampling with replacement from the respondents of the RDS study with sampling weight 1/w_i_, where w_i_ is the current weight estimate. The list of all possible heterophilic links was then enumerated by considering all pairs with mismatching serostatus according to node degree. The required number of heterophilic links (HL =p*(1-p)* d_HIV+_* d_HIV-_)*N/ d_all_*H where d_X_ is the average degree in population X, p prevalence and H homophily) were then taken at random in this list. The network was then be completed by randomly connecting the remaining available slots among individuals with matching serostatus. This simple approach ensures that the simulated network agrees with the proposed characteristics.
